# Thromboembolism in patients with melanoma receiving immune checkpoint inhibitors: incidence and risk factors

**DOI:** 10.1016/j.iotech.2025.101063

**Published:** 2025-06-21

**Authors:** D.C.H. van Dorst, M.M. Hofman, R.M. de Waal, E. Oomen-de Hoop, K. de Joode, S. Bins, S.L.W. Koolen, A. Joosse, J. Versmissen, M.J.H.A. Kruip, A.A.M. van der Veldt, R.H.J. Mathijssen

**Affiliations:** 1 Department of Medical Oncology, Erasmus University Medical Center, Cancer Institute, Rotterdam, The Netherlands; 2 Division of Vascular Medicine and Pharmacology, Department of Internal Medicine, Erasmus University Medical Center, Rotterdam, The Netherlands; 3 Department of Pulmonary Medicine, Erasmus University Medical Center, Rotterdam, The Netherlands; 4 Department of Hospital Pharmacy, Erasmus University Medical Center, Rotterdam, The Netherlands; 5 Department of Hematology, Erasmus University Medical Center, Rotterdam, The Netherlands

**Keywords:** melanoma, immune checkpoint inhibitors, thromboembolism, arterial thromboembolism, venous thromboembolism, side-effects

## Abstract

**Background:**

Immune checkpoint inhibitors (ICIs) may increase the incidence of thromboembolisms (TEs). In patients with melanoma, the risk of TE and its association with the disease setting remains largely unknown. This study investigated the incidences of arterial thromboembolism (ATE) and venous thromboembolism (VTE) and associated risk factors in ICI-treated patients with melanoma in the advanced and adjuvant disease settings.

**Patients and methods:**

ATE and VTE incidences were retrospectively collected from patients receiving ICI in the advanced (irresectable stage III/IV) or adjuvant (stage III/IV, after complete resection) disease setting until 2 years or after censoring. Associations between characteristics and ATE/VTE were analyzed using the Fine–Gray model, with all-cause death as competing risk.

**Results:**

In the advanced disease cohort, 7 (2.2%) and 16 (5.1%) of 315 included patients developed ATE and VTE, respectively. In the adjuvant cohort, 3 (2.1%) and 2 (1.4%) of 143 included patients developed ATE and VTE, respectively. In the advanced cohort, ICI combination therapy was associated with VTE [standardized hazard ration (sHR) 4.42, 95% confidence interval (CI) 1.58-12.38, *P* = 0.005] and higher body mass index (BMI) with ATE (sHR 1.09 per point BMI increase, 95% CI 1.02-1.17, *P* = 0.017), with a trend toward VTE (sHR 1.06, 95% CI 1.00-1.13, *P* = 0.055). In the adjuvant cohort, VTE history was associated with increased combined TE (sHR 11.22, 95% CI 1.83-68.68, *P* = 0.009) and higher BMI with a trend toward increased TE (sHR 1.19, 95% CI 0.97-1.46, *P* = 0.091).

**Conclusions:**

Both ATE and VTE incidences seem to be modest in patients with melanoma receiving ICI in both disease settings. Patients with higher BMI and who are prescribed ICI combination therapy might be at increased thrombotic risk.

## Highlights

• ATE and VTE incidence in melanoma patients was analyzed separately for advanced and adjuvant ICI therapy.
• Incidences of both ATE and VTE were relatively modest in both treatment settings.
• Patients who receive ICI combination therapy and with a higher BMI have a relatively higher thrombotic risk.
• Extra monitoring in high-risk patients and further research on the role of tumor burden and combination therapy are required.

## Introduction

Immune checkpoint inhibitors (ICIs) have revolutionized the therapeutic landscape of a variety of tumor types, frequently improving overall survival and even leading to curation in a subset of patients.^1^ ICIs are monoclonal antibodies which induce an immune-mediated antitumor response by inhibition of immunosuppressive immune checkpoints, including the cytotoxic T lymphocyte antigen-4 (CTLA-4) receptor and the programmed cell death protein 1 (PD-1) receptor or its ligand (PD-L1).^1^ Despite their efficacy in a substantial proportion of patients, ICI treatment is often hampered by immune-related adverse events which can affect every organ system including the cardiovascular system.^2^ Serious concerns have arisen that ICIs may also increase the incidences of both arterial and venous thromboembolisms (ATE and VTE, respectively), which are collectively referred to as thromboembolisms (TEs).^3^^,^^4^ The occurrence of TEs can negatively impact the quality of life and adversely affects survival outcomes in patients with cancer.^5^^,^^6^

To date, the exact mechanisms by which ICI may lead to both ATE and VTE remain largely unknown. Both preclinical and clinical studies point toward proatherosclerotic effects of ICIs^7^^,^^8^ and ICI-induced vasculitis in rare cases,^9^ which could directly predispose patients to ATEs in particular. These proatherosclerotic effects and increased ATE risk during ICI therapy were also reported in a large clinical study.^10^ Moreover, ICI-associated mechanisms which could predispose to VTE in particular include increased production of the procoagulant tissue factor by T-cell-mediated monocyte activation and the release of proinflammatory cytokines leading to endothelial activation and prothrombotic immune cell activation.^11^^,^^12^

ICI therapy is frequently administered sequentially and/or concurrent with other anticancer treatments with prothrombotic effects including chemotherapy and radiotherapy.^13^^,^^14^ In addition, patients with cancer already have a higher risk of developing TE compared with the general population.^15^ Therefore, the assessment of the specific contribution of ICI therapy to TE is challenging, which may explain conflicting results in previous studies.^4^^,^^16^^,^^17^ In addition, most previous studies have included various tumor types in both early and advanced stages, whereas both tumor type and stage of disease are independent determinants for TE occurrence after cancer diagnosis independent of the administered therapy.^18^^,^^19^ Therefore, the effects of ICI therapy on TE incidence should be studied in a tumor-specific manner with additional consideration of the stage of the included tumor type. In patients with melanoma, ICIs are administered in the advanced disease (irresectable stage III or stage IV) and in the adjuvant disease (stage III or IV, after complete resection) setting. This broad application and the administration of ICIs in the adjuvant setting with curative intent necessitate adequate monitoring of both short- and longer-term adverse events, including TE. Until now, only a single retrospective study investigated the occurrence of TE during ICI therapy in patients with melanoma specifically, reporting a TE incidence of 9.3% and 16% after 6 months and 12 months, respectively.^20^ Yet, >80% of included patients in this study had stage IV melanoma and no distinction was made between the advanced and adjuvant setting.^20^ Therefore, in the current study, we investigated the incidences and risk factors for VTE and ATE during ICI therapy in patients with melanoma who were treated with ICIs in both the advanced disease and the adjuvant disease settings separately.

## Patients and methods

### Patient population

In the current retrospective cohort study, patients with melanoma who received anti-PD-1 monotherapy (nivolumab or pembrolizumab), anti-CTLA-4 monotherapy (ipilimumab), or anti-PD-1/anti-CTLA-4 combination (nivolumab + ipilimumab) therapy as standard of care were included. Patients were categorized into either the adjuvant (stage III and IV, after complete resection) or advanced (irresectable stage III or stage IV) disease setting based on their stage at baseline. Patients were not restaged during this study (e.g. in case of recurrence or progression) and were excluded if they had previously received ICI treatment. The current retrospective cohort study was a secondary analysis from patients who were included between 5 April 2016 and 9 September 2021 in the prospective MULTOMAB study conducted at the Erasmus MC University Medical Center (International Clinical Trials Registry Platform, NTR7015).^21^ The aim of the MULTOMAB study is to establish a prospectively collected database comprising both clinical data and blood samples from oncology patients treated with monoclonal antibodies, including ICI treatment, in order to investigate treatment effectiveness and associated side-effect. The study was approved by the Medical Ethics Committee of the Erasmus University Medical Center (MEC 16-011). All patients provided written informed consent before participation and the study was conducted according to the Declaration of Helsinki.

### Endpoints

The primary endpoints of this study were the incidences of ATE and VTE within 2 years after the start of ICI administration or until censoring in the advanced disease and adjuvant cohorts separately. ATE was defined as the occurrence of any of the following events: myocardial infarction, coronary artery disease requiring revascularization, ischemic stroke, transient ischemic attack requiring medical intervention, and peripheral arterial thrombosis/embolism. VTE was defined as the occurrence of any of the following events: deep venous thrombosis (DVT), pulmonary embolism (PE), cerebral venous sinus thrombosis, and visceral vein thrombosis. Cases of thrombophlebitis were excluded as a VTE. For ATE and VTE, both symptomatic and asymptomatic events were included. The secondary aim of this study was to identify potential risk factors for the development of TE after start of ICI treatment. Risk factors for both ATE and VTE were analyzed separately if substantial numbers of both types of TE were observed.

Follow-up was stopped if a TE occurred and patients were censored (i.e. data collection and screening for the incidence of TE was stopped from that specific time point) in case of a switch to alternative anticancer treatment (e.g. BRAF/MEK inhibitors), or loss to follow-up, or switch to a different ICI treatment regimen initiated >3 months after the last treatment of the initial ICI regimen (e.g. switch from nivolumab to combination nivolumab/ipilimumab). Events were identified through manual chart review of the electronic medical record. An event was classified as a myocardial infarction in case medical therapy was initiated and as a requirement of coronary vascularization in case the revascularization was carried out. An ischemic stroke/TIA was only included as an ATE in case medical therapy (e.g. anticoagulation) was initiated. All VTE were previously diagnosed using radiological imaging. All events were verified by two independent reviewers (DCHvD and MMH).

### Data collection

The following characteristics were retrospectively collected from the electronic medical records at baseline (or if not available at baseline within 3 months prior): age, sex, World Health Organization (WHO) performance status, body mass index (BMI), smoking status (tobacco use only; excluding vapes/e-cigarettes and smokers were categorized into patients who ever smoked (current + former) and patients who never smoked, medication use, melanoma stage, lactate dehydrogenase (LDH) levels, presence of brain metastases, type of ICI treatment, usage of corticosteroids, previous anticancer treatment, previous history of ATE and VTE, Khorana risk score,^22^ recent hospitalization (admission of at least one night for any reason within 1 month before baseline), and recent surgery (≤3 months before baseline). Baseline was defined as the date of ICI therapy initiation. Data cut-off was set at 1 December 2022.

### Statistical analysis

Continuous variables were reported as mean and standard deviation or median and interquartile range (IQR). Categorical variables were described as counts and percentages. Patient and clinical characteristics were compared between the advanced and adjuvant cohort with the Mann–Whitney *U* test, Pearson X^2^ test, or Fisher’s exact test. The primary endpoints of the study were the cumulative incidence rates of ATE and VTE per 100 person-years (PY) of follow-up for the advanced disease and adjuvant cohorts separately.

For the secondary endpoint, the association between patient and clinical characteristics and (the time to) ATE or VTE occurrence was studied by means of the proportional hazard model for the subdistribution as described by Fine and Gray,^23^ where all-cause death before TE was regarded as competing risk. Effect estimates are presented as subdistribution hazard ratios (sHRs) with 95% confidence intervals (CIs). Parameters with a *P* value < 0.10 in the univariable analysis were selected and tested in a multivariable proportional hazard model for the subdistribution. Multicollinearity is evident among the presence of brain metastases at baseline, ICI combination therapy, and LDH. Therefore, in case of *P* < 0.10 for multiple of these variables in the univariable analysis, only the variable with the lowest *P* value was included in the multivariable analysis. To assess whether the occurrence of ATE or VTE was associated with survival, a Cox proportional hazards model with the occurrence of ATE and VTE as time-dependent variable was used [i.e. intervals of time were coded with the first interval beginning at start of treatment until occurrence of either ATE or VTE (whichever occurred first) and the second interval ranging from this moment until death from any cause or end of follow-up or censoring]. Data were analyzed using SPSS Statistics (IBM, version 28) and Stata (StataCorp. 2021. Stata: Release 17.1 Statistical Software; StataCorp LP, College Station, TX). All *P* values were two-sided, confidence intervals were set at the 95% level, and *P* values < 0.05 were considered to indicate statistical significance.

## Results

### Baseline characteristics

A total of 458 patients were included in this study, of whom 315 patients received ICI treatment for irresectable stage III or stage IV melanoma (advanced cohort) and 143 patients after resected stage III or IV melanoma (adjuvant cohort). Baseline characteristics are displayed in Table 1. In both the advanced and adjuvant cohorts, median age was 64 years and the majority of included patients were male (58% and 63%, respectively). In the advanced cohort, 287 (91%) patients had melanoma stage IV and 66 (21%) of 315 patients had brain metastases at baseline. Moreover, in this cohort, anti-PD-1 monotherapy (nivolumab or pembrolizumab) (67%) and anti-PD-1 + anti-CTLA-4 combination therapy (nivolumab + ipilimumab) (32%) were the most commonly prescribed ICI treatments. The adjuvant cohort predominantly consisted of patients with melanoma stage III (94%), of which all patients received anti-PD-1 monotherapy. All patients in the adjuvant cohort underwent surgical removal of the tumor. In 127 (89%) of the patients, this took place within 3 months before the start of ICI therapy. Median time between surgery and initiation of ICI therapy was 10.1 weeks (IQR 8.4-12.5 weeks). Patients in the advanced disease cohort had a worse WHO performance status at baseline than patients in the adjuvant cohort (*P* = 0.003). More patients in the advanced disease cohort received prior targeted therapy (14% versus 1%, *P* < 0.001) or prior radiotherapy (11% versus 2%, *P* < 0.001). More patients in the advanced disease cohort had a history of VTE compared with patients in the adjuvant cohort (9% versus 3%, *P* = 0.026).

**Table 1:** Baseline characteristics of the study population

	Advanced disease cohort (*n* = 315)	Adjuvant cohort (*n* = 143)	*P* value
Demographics			
Age at treatment start, years, median (IQR)	64 (54-72)	64 (57-73)	0.56^a^
Sex, *n* (%)			0.41^b^
Male	184 (58)	90 (63)	
Female	131 (42)	53 (37)	
Smoking status			0.25^c^
Non-smoker	155 (49)	61 (43)	
Former smoker	94 (30)	54 (38)	
Current smoker	34 (11)	18 (13)	
Missing	32 (10)	10 (7)	
WHO performance status, *n* (%)			**0.003**^c^
0	184 (58)	105 (73)	
1	103 (33)	26 (18)	
2	9 (3)	2 (1)	
Missing	19 (6)	10 (7)	
BMI (kg/m^2^), median (IQR)	27 (24-30)	27 (24-30)^d^	0.49^a^
Baseline eGFR < 60 (ml/min/1.73 m^2^), *n* (%)	44 (14)	15 (11)	0.37^b^
Cancer diagnosis, *n* (%)			
Melanoma stage			**<0.001**^b^
III, resectable	NA	135 (94)	
III, irresectable	28 (9)	NA	
IV	287 (91)	8 (6)	
Brain metastases at treatment start	66 (21)	NA	
Elevated LDH (>1× ULN), *n* (%)	125 (40)^e^	14 (10)^f^	**<0.001**^b^
Treatment characteristics, *n* (%)			
Prior anticancer therapy			
Prior targeted therapy	43 (14)	2 (1)	**<0.001**^b^
Prior chemotherapy	5 (2)	0 (0)	0.33^b^
Prior radiotherapy	36 (11)	3 (2)	**<0.001**^c^
Recent surgery (<3 months)	48 (15)	127 (89)	**<0.001**^c^
First administered ICI			**<0.001**^c^
Nivolumab	156 (50)	123 (86)	
Pembrolizumab	54 (17)	20 (14)	
Ipilimumab	3 (1)	0 (0)	
Nivolumab + ipilimumab	102 (32)	0 (0)	
History of thromboembolic events, *n* (%)			
History of ATE	36 (11)	14 (10)	0.75^b^
History of VTE	27 (9)	4 (3)	**0.026**^b^
TE-related factors, *n* (%)			
Khorana risk score at treatment start			**<0.001**^b^
0	214 (68)	124 (87)	
≥1	101 (32)	19 (13)	
Recent hospitalization (<30 days)	29 (9)	2 (1)	**0.005**^b^
Medication use at ICI treatment start, *n* (%)			
Anticoagulants	67 (22)	31 (22)	0.051^b^
VKA	13 (4)	1 (1)	
DOAC	11 (3)	8 (6)	
LMWH	11 (3)	1 (1)	
Antiplatelet agents	30 (10)	21 (15)	
Combination (>1)	2 (1)	0 (0)	
Antihypertensive	122 (39)	63 (44)	0.31^b^
Antihyperlipidemic	71 (23)	41 (29)	0.16^b^
Antidiabetic	4 (11)	12 (8)	0.50^b^
Corticosteroids	15 (5)	0 (0)	**0.004**^b^

*P* values < 0.05 are indicated in bold.

ATE, arterial thromboembolism; BMI, body mass index; DOAC, direct oral anticoagulant; eGFR, estimated glomerular infiltration rate; ICI, immune checkpoint inhibitor; IQR, interquartile range; LDH, lactate dehydrogenase; LMWH, low-molecular-weight heparin; NA, not applicable; TE, thromboembolism; ULN, upper limit of normal; VKA, vitamin K antagonist; VTE, venous thromboembolism; WHO, World Health Organization.

a Mann–Whitney *U* test.

b Fisher’s exact test.

c Chi-square test.

d BMI was missing for one patient.

e LDH was missing for three patients.

f LDH was missing for two patients.

### Incidence and characteristics of thromboembolic events

After a median follow-up duration of 16.8 months (IQR 5.8-24.0 months), a total of 10 ATEs and 18 VTEs were observed in the total cohort. These occurred after a median duration of 9.1 months (IQR 3.0-17.6 months) and 4.5 months (IQR 2.6-11.8 months) after the start of ICI therapy, respectively. Table 2 displays the incidences of ATE, VTE, and total TE for the advanced and adjuvant cohorts separately. Cumulative incidence functions of ATE and VTE and detailed characteristics of the individual TE events are summarized in Supplementary Figure S1 and Table S1, available at https://doi.org/10.1016/j.iotech.2025.101063, respectively. Four out of the total 28 patients who experienced a TE were hospitalized during the event, possibly indicating immobilization as a factor contributing to the event. Of note, only one patient who experienced a stroke was diagnosed with atrial fibrillation at the time of the event, for which anticoagulation was initiated afterwards. In another patient who experienced a stroke but did not have atrial fibrillation, a floating thrombus in the aorta was detected at the time of the event.

**Table 2:** Incidence of thromboembolic events and incidence rates

	Advanced disease cohort (*n* = 315)	Adjuvant cohort (*n* = 143)
Events, *n* (%)		
ATE	7 (2.2)	3 (2.1)
VTE	16 (5.1)	2 (1.4)
Total TE	23 (7.3)	5 (3.5)
Incidence rate [per 100 person-years (95% CI)]		
ATE	1.9 (0.8-3.8)	1.5 (0.4-4.0)
VTE	4.4 (2.6-7.0)	1.0 (0.2-3.2)
Total TE	6.4 (4.1-9.4)	2.4 (0.9-5.4)
Time to occurrence [median (IQR), months]		
ATE	9.9 (3.2-21.2)	5.1 (2.6-17.2)
VTE	4.5 (2.6-9.4)	10.3 (2.5-NA)^a^

ATE, arterial thromboembolism; CI, confidence interval; IQR, interquartile range; NA, not assessable; TE, thromboembolism; VTE, venous thromboembolism.

a Median time to occurrence is based on only two events in total.

#### Advanced disease cohort

In the advanced disease cohort specifically, median follow-up was 13.8 months (IQR 4.0-24.0 months). A total of 118 out of 315 (37.5%) patients died within 2 years after start of ICI therapy. Twenty-three (7.3%) of the 315 included patients with advanced disease developed a TE, which occurred after a median duration of 5.3 months (IQR 2.6-12.3 months) after start of ICI therapy. This corresponded with a total TE incidence rate of 6.4 per 100 PY (95% CI 4.1-9.4 per 100 PY). Of these events, 17 were symptomatic and 6 were incidental (i.e. asymptomatic and detected on routine imaging; Supplementary Table S1, available at https://doi.org/10.1016/j.iotech.2025.101063).

Seven (30%) of 23 observed TEs were ATE, which corresponded to an absolute ATE incidence of 2.2% and an incidence rate of 1.9 per 100 PY (95% CI 0.8-3.8 per 100 PY). ATEs consisted of stroke (*n* = 4), transient ischemic attack (*n* = 1), myocardial infarction (*n* = 1), and the requirement of coronary revascularization (*n* = 1). Sixteen (70%) of 23 observed TEs were VTE, which corresponded to an absolute VTE incidence of 5.1% and an incidence rate of 4.4 per 100 PY (95% CI 2.6-7.0 per 100 PY). VTEs consisted of PE (*n* = 11), a combination of PE and DVT (*n* = 3), DVT (*n* = 1), and cerebral venous thrombosis (*n* = 1). The median duration until occurrence was longer for ATE [9.9 months (IQR 3.2-21.2 months)] than for VTE [4.5 months (IQR 2.6-9.4 months)].

#### Adjuvant cohort

In the adjuvant cohort, median follow-up was 17.8 months (IQR 12.9-24.0 months). In total, 5 (3.5%) of the 143 patients in the adjuvant cohort developed a TE, which occurred after a median duration of 5.1 months (IQR 2.6-17.2 months) after start of ICI therapy. This corresponded with a total TE incidence rate of 2.4 per 100 PY (95% CI 0.9-5.4 per 100 PY). All events were symptomatic. Patients with a TE had no evidence of cancer recurrence at the time of event, nor did they experience ICI-related toxicity of grade ≥ 3.

Three (60%) of five observed TEs were ATE, which corresponded to an absolute ATE incidence of 2.1% and an incidence rate of 1.5 per 100 PY (95% CI 0.4-4.0 per 100 PY). ATE consisted of stroke (*n* = 2) and myocardial infarction (*n* = 1). Two (40%) of five observed TEs were VTE, which corresponded to an absolute VTE incidence of 1.4% and an incidence rate of 1.0 per 100 PY (95% CI 0.2-3.2 per 100 PY). VTE consisted of PE (*n* = 1) and DVT (*n* = 1). Of note, the patient who developed a DVT had an inherited prothrombotic condition (heterozygote Factor V Leiden) and did not use anticoagulation. The patient who developed a PE was also diagnosed with renal cell carcinoma and experienced the event shortly after a nephrectomy. During this procedure, the previously prescribed antiplatelet therapy (indication: primary cardiovascular prevention) was discontinued temporarily, but this patient received adequate thrombosis prophylaxis.

### Risk factors for TE during ICI therapy

#### Advanced cohort

In the advanced disease cohort, the association between clinical parameters and the occurrence of ATE (Table 3) and VTE (Table 4) were assessed separately. Supplementary Table S2, available at https://doi.org/10.1016/j.iotech.2025.101063, summarizes risk factors for combined TE occurrence.

**Table 3:** Competing risk regression for the occurrence of ATE in the advanced disease cohort

	Univariable			
	*n* (%)	sHR	95% CI	*P* value
Age (≥65 years)	155 (49)	2.40	0.46-12.46	0.30
Sex (female)	131 (42)	1.12	0.26-4.87	0.88
Smoking (ever versus never)	128 (41)	2.79	0.54-14.45	0.22
WHO (≥1)	112 (36)	0.68	0.13-3.50	0.65
BMI	Continuous	1.09	1.02-1.17	0.017
LDH (>1 ULN)	125 (40)	1.13	0.25-5.03	0.87
Brain metastases	66 (21)	1.67	0.31-8.93	0.55
ICI combination therapy	102 (32)	0.44	0.05-3.54	0.44
History of ATE	36 (11)	1.16	0.14-9.43	0.89
Khorana risk score at treatment start (≥1)	101 (32)	0.80	0.16-4.08	0.80
Recent surgery	48 (15)	1.00	0.12-8.47	1.00
Anticoagulant therapy (any)	67 (21)	1.39	0.27-7.16	0.70
Antihypertensive therapy	122 (39)	2.00	0.47-8.59	0.35
Antihyperlipidemic therapy	71 (23)	1.29	0.26-6.51	0.75

History of VTE could not be added to the model because no patient with a history of VTE experienced an ATE.

ATE, arterial thromboembolism; BMI, body mass index; CI, confidence interval; ICI, immune checkpoint inhibitor; LDH, lactate dehydrogenase; sHR, subdistribution hazard ratio; ULN, upper limit of normal; VTE, venous thromboembolism; WHO, World Health Organization.

**Table 4:** Competing risk regression for the occurrence of VTE in the advanced disease cohort

	Univariable				Multivariable		
	*n* (%)	sHR	95% CI	*P* value	sHR	95% CI	*P* value
Age (≥65 years)	155 (49)	0.58	0.21-1.61	0.29			
Sex (female)	131 (42)	1.14	0.42-3.06	0.80			
Smoking (ever versus never)	128 (41)	0.39	0.13-1.21	0.10			
WHO (≥1)	112 (36)	1.04	0.37-2.88	0.94			
BMI	Continuous	1.05	1.00-1.11	0.071	1.06	1.00-1.13	0.055
LDH (>1 ULN)	125 (40)	1.54	0.58-4.09	0.40			
Brain metastases	66 (21)	2.60	0.94-7.20	0.065			
ICI combination therapy	102 (32)	4.29	1.54-11.95	0.005	4.42	1.58-12.38	0.005
History of VTE	27 (9)	0.73	0.10-5.35	0.76			
History of ATE	36 (11)	1.65	0.47-5.83	0.44			
Khorana risk score at treatment start (≥1)	101 (32)	0.68	0.22-2.14	0.51			
Recent surgery	48 (15)	0.83	0.19-3.58	0.80			
Recent hospitalization	29 (9)	1.50	0.33-6.73	0.60			
Anticoagulant therapy (any)	67 (22)	1.18	0.38-3.68	0.77			

ATE, arterial thromboembolism; BMI, body mass index; CI, confidence interval; ICI, immune checkpoint inhibitor; LDH, lactate dehydrogenase; sHR, subdistribution hazard ratio; ULN, upper limit of normal; VTE, venous thromboembolism; WHO, World Health Organization.

BMI was the only variable associated with the occurrence of ATE in the univariable analysis (sHR 1.09 per one-point increase in BMI, 95% CI 1.02-1.17, *P* = 0.017) (Table 3). Usage of anticoagulant therapy was not associated with ATE (sHR 1.39, 95% CI 0.27-7.16, *P* = 0.70).

Higher BMI (sHR 1.05 per point BMI increase, 95% CI 1.00-1.11, *P* = 0.071), ICI combination therapy (sHR 4.29 versus monotherapy, 95% CI 1.54-11.95, *P* = 0.005), and the presence of brain metastases at baseline (sHR 2.60, 95% CI 0.94-7.20, *P* = 0.065) were associated with the occurrence of VTE in the univariable analysis (Table 4). Usage of anticoagulant therapy was not associated with VTE (sHR 1.18, 95% CI 0.38-3.68, *P* = 0.77).

The variable ‘brain metastases’ was not included in the multivariable model due to multicollinearity with receiving ICI combination therapy [i.e. combination therapy was given to 23% of the patients without brain metastases versus 67% of those with brain metastases (*P* < 0.001)]. In the multivariable analysis, ICI combination therapy (sHR 4.42 versus monotherapy, 95% CI 1.58-12.38, *P* = 0.005) remained associated with the occurrence of VTE. In line with this, the incidence rate of VTE was substantially higher in patients receiving ICI combination therapy compared with monotherapy: 9.9 per 100 PY (95% CI 5.0-17.7 per 100 PY) versus 2.3 per 100 PY (95% CI 0.9-4.8 per 100 PY), respectively (Supplementary Table S3, available at https://doi.org/10.1016/j.iotech.2025.101063). Higher BMI demonstrated a trend toward increased VTE occurrence (sHR 1.06 per point BMI increase, 95% CI 1.00-1.13, *P* = 0.055).

#### Adjuvant cohort

Due to the limited number of events in the adjuvant cohort, the association between clinical parameters and the overall occurrence of TE (combined ATE and VTE) was assessed in this cohort (Table 5). A history of VTE was associated with the occurrence of TE in the univariable analysis (sHR 11.22, 95% CI 1.83-68.68, *P* = 0.009). BMI demonstrated a trend toward higher TE occurrence (sHR 1.19 per point increase, 95% CI 0.972-1.458, *P* = 0.091). The clinical parameters LDH and Khorana risk score could not be tested in the univariable model due to the limited number of events per subgroup. Usage of anticoagulant therapy was not associated with TE (sHR 2.49, 95% CI 0.41-15.14, *P* = 0.32). Due to the low number of events, no multivariable analysis was carried out in the adjuvant cohort. In the total cohort, there was no difference in overall survival between patients who experienced a TE versus those who did not (HR 1.38, 95% CI 0.67-2.84, *P* = 0.38).

**Table 5:** Competing risk regression for the occurrence of combined TE in the adjuvant cohort

	*N* (%)	sHR	95% CI	*P* value
Age (≥65 years)	69 (48)	0.71	0.12-4.17	0.71
Sex (female)	53 (37)	1.26	0.21-7.57	0.80
Smoking (ever versus never)	72 (50)	1.31	0.22-7.66	0.77
WHO (≥1)	28 (20)	1.22	0.14-11.07	0.86
BMI	Continuous	1.19	0.97-1.46	0.091
Recent surgery (yes versus no)	127 (89)	0.44	0.50-3.94	0.47
History of ATE	14 (10)	2.31	0.26-20.76	0.46
History of VTE	4 (3)	11.22	1.83-68.68	0.009
Anticoagulant therapy (any)	31 (22)	2.49	0.41-15.14	0.32

ATE, arterial thromboembolism; BMI, body mass index; CI, confidence interval; sHR, subdistribution hazard ratio; TE, thromboembolism; VTE, venous thromboembolism; WHO, World Health Organization.

## Disussion

### Arterial thromboembolism

In this real-world cohort study, we found that 2% of patients with melanoma developed an ATE within 2 years of initiation of ICI therapy in both the advanced disease and adjuvant disease settings. Our ATE incidence is similar to a previous study that reported an incidence of 1.8% in ICI-treated patients with various underlying malignancies including melanoma.^4^ To the best of our knowledge, the exact ATE incidence in patients with melanoma without ICI therapy is unknown, which hinders the interpretation of the specific contribution of ICI to ATE risk in our study. None the less, individuals aged 65-75 years without cancer already have a 1.4% yearly incidence of ATE, which is increased by 1.5-fold to 2.2% in case cancer is present.^24^ Moreover, the general incidence of ATE is ∼2%-6% within 1 year of cancer diagnosis.^25^ Therefore, the risk of ATE does not seem to be substantially higher in our study population than the general population of patients with cancer. This indicates that ICIs do not significantly increase ATE risk within the first 2 years of therapy.

### Venous thromboembolism

We found that the incidences of VTE were higher in the advanced disease cohort (5.1%) compared with the adjuvant cohort (1.4%), which corresponded to a VTE incidence rate of 4.4 and 1.0 per 100 PY, respectively. These respective VTE incidences were similar to the incidences observed previously in patients with advanced melanoma (4.4 per 100 PY)^26^ and patients with regional melanoma (0.7 per 100 PY) who did not receive ICI therapy.^26^^,^^27^ Moreover, the two patients with VTE in the adjuvant cohort were at increased thrombotic risk due to other reasons (heterozygote factor V Leiden and occurrence of post-operative thrombosis). In line with a systematic review article on VTE incidence in ICI-treated patients with various underlying malignancies,^16^ our results therefore strongly indicate that ICIs do not directly increase VTE risk compared with other anticancer agents used for the treatment of melanoma.

### Comparison of ATE and VTE incidences with previous literature

Our observed incidences of ATE and VTE are comparable to most previous studies, with the exception of Sussman et al.^20^ and Drobni et al.^10^ Sussman et al. report substantially higher incidence rates of ATE (6.1%) and VTE (16.2%) in their cohort of patients with melanoma within 1 year of initiation of ICI therapy.^20^ Of note, their cohort was smaller (*n* = 228) and consisted of a larger proportion of stage IV patients who generally have a higher thrombotic risk. In addition, previous anticancer therapies with possible prothrombotic effects^28^ were not specified by the authors, which complicates the exact comparison with our study. Drobni et al.^10^ report a higher incidence rate of ATE as well (6.55 per 100 PY, compared with 1.9 and 1.5 per 100 PY in our advanced disease and adjuvant cohorts, respectively). Although patient characteristics were generally comparable to patients included in our study, only 28% of their study population consisted of patients with melanoma. Given that melanoma is a tumor type with relatively low thrombogenic potential,^26^ this could explain their substantially higher reported ATE incidence.

### Risk factors for thromboembolisms

We found that higher BMI was significantly associated with increased ATE risk and a trend toward increased VTE risk in the advanced disease cohort. Furthermore, higher BMI demonstrated a trend toward increased TE risk in the adjuvant cohort as well. Higher BMI is an established risk factor for both ATE and VTE in the general population^29^ and our results are in line with a study carried out in lung cancer patients that reports very high BMI (≥40 kg/m^2^) as a risk factor for VTE during ICI therapy.^30^ Moreover, we found that ICI combination therapy was associated with increased VTE risk. Yet, this combinatory regimen is more frequently administered to patients with the highest disease burden or with brain metastases who already have an increased risk of thrombosis.^19^ Therefore, this finding could be subjected to confounding by indication (i.e. the higher disease burden predisposing these patients to thrombosis rather than ICI combination therapy specifically). We further examined the contribution of a higher disease burden via LDH levels, but found no association between higher LDH levels at baseline and either ATE or VTE occurrence. In addition, compared with patients in the adjuvant setting, VTE incidence rates were only slightly higher in advanced disease patients receiving monotherapy (1.0 versus 2.3 per 100 PY, respectively), but substantially higher in patients receiving combination therapy (9.9 per 100 PY). Therefore, the possible contribution of ICI combination therapy on VTE incidence in particular requires extra investigation. Although ICI therapy has been associated with atrial fibrillation,^31^ only one patient who experienced a stroke had atrial fibrillation during the event. Therefore, a major contributing role of ICI-induced atrial fibrillation in thrombosis is unlikely in our study.

### Limitations

Our study is the first to analyze the incidences of ATE and VTE in a real-world cohort of patients with melanoma in the advanced disease and adjuvant disease settings separately, but has some limitations. Firstly, we did not have a control cohort of patients with melanoma who did not receive ICI therapy. Therefore, we had to compare our ATE and VTE incidences with incidences from previous studies. Secondly, a substantial number of patients were lost to follow-up within 2 years of inclusion due to its retrospective design. We corrected for this loss of follow-up by censoring and calculation of TE incidence per 100 PY. Thirdly, because of our relatively short follow-up period, we could not determine the longer-term effects of ICI treatment on thrombotic risk. Given that ICIs are demonstrated to stimulate atherosclerosis and that clinically significant atherosclerotic lesions may take years to develop,^7^^,^^8^ it would be particularly interesting to monitor the incidence of ATE in the longer term. Fourthly, despite the fact that our cohort was twice the size of the previous study,^20^ the number of events observed in our study was relatively limited. Although this could indicate that ATE and VTE are not very frequently encountered adverse events in melanoma patients receiving ICI, we could not assess factors associated with ATE and VTE separately in the adjuvant cohort. Further studies in larger groups of patients receiving ICI in the adjuvant setting are required. Lastly, due to its retrospective design, all included patients on anticoagulation had a preexisting indication for this therapy (e.g. history of TE). Therefore, we could not adequately investigate the possible preventive effects of anticoagulation on thrombosis during ICI therapy. Future prospective studies should provide more insights on the possible preventive effects of prophylactic anticoagulation strategies on thrombosis in ICI-treated patients with the highest risk.

In conclusion, the risk of developing ATE is similar but low in patients with melanoma receiving ICI therapy in the advanced disease and adjuvant disease settings. Although VTE was more prevalent in the advanced disease cohort, the observed VTE incidences were comparable to cohorts of patients who did not receive ICI treatment in previous studies. Therefore, our findings provide a first indication that ICI therapy does not directly increase thrombotic risk in patients with melanoma. We found that thrombosis was more common in patients with higher BMI and who are prescribed ICI combination therapy. This suggests that extra monitoring and patient information might be required in this group. Future prospective studies should provide more insights into the possible individual roles of ICI combination therapy, tumor burden, and the presence of brain metastases as possible independent thrombotic risk factors and in the longer-term effects of ICI treatment on thrombotic risk.
