# Supplementary material for: Thromboembolism in patients with melanoma receiving immune checkpoint inhibitors: incidence and risk factors
Source: Immunooncol Technol. 2025 Jun 21;27:101063. doi: 10.1016/j.iotech.2025.101063 (PMC12275875; doi:10.1016/j.iotech.2025.101063)
Supplement: Supplementary Data [file mmc1.docx]

**Thromboembolism in patients with melanoma receiving immune checkpoint inhibitors: incidence and risk factors**

**Central illustration**………………………..…………………………………………Page 2

**Supplemental Figure 1:** Cumulative incidence functions of thromboembolic events in the advanced disease and adjuvant cohorts……………………………….. ……………..Page 3

**Supplemental Table 1:** Description of thromboembolic events observed in patients with melanoma receiving ICI in the advanced disease and adjuvant cohorts ……....……..Page 4-7

**Supplemental Table 2:** Competing-risk regression for the occurrence of combined TE in the advanced disease cohort….. ………………………………………………………….Page 8

**Supplemental Table 3:** Incidence of thromboembolic events and incidence rates in the advanced disease cohort………………………………………………………………Page 9

**Central illustration: Incidence and risk factors for arterial and venous thromboembolism in patients with melanoma receiving immune checkpoint inhibitor therapy.**

Advanced disease indicates irresectable stage III or stage IV disease. Adjuvant disease indicates stage III or stage IV after complete resection. Abbreviations: BMI, body mass index; ICI, immune checkpoint inhibitor; sHR, subdistribution hazard ratio VTE, venous thromboembolism. Values indicate 95% confidence intervals. Indicated sHRs for VTE in the advanced disease cohort and total TE in the adjuvant cohort display results of the multivariable analyses.

*sHR is reported per one-point increase in BMI

**Supplemental Figure 1.** Cumulative incidence function of (**A)** arterial thromboembolism (ATE) and (**B**) venous thromboembolism (VTE) in the advanced disease cohort (n=315), and (**C**) ATE and (**D**) VTE in the adjuvant cohort (n=143). Only the first thrombotic event per patient was taken into account. Median follow-up was 13.8 [IQR 4.0-24.0] months in the advanced disease cohort, and 17.8 [IQR 12.9-24.0] months in the adjuvant cohort, respectively.

**Supplemental Table 1. Description of thromboembolic events observed in patients with melanoma receiving ICI in the advanced disease and adjuvant cohorts**

| Patient number | Age | Sex (M/F) | TE  (type) | Brain metastases (at moment of TE) | Treatment setting | ICI mono vs. combi | BMI  (kg/m^2^) | ATE or VTE  history | Prior treatment | Anticoagulant use BL? | TE symptomatic? | Hospitalization required due to TE? | Remarks |
| --- | --- | --- | --- | --- | --- | --- | --- | --- | --- | --- | --- | --- | --- |
| 1 | 59 | F | PE | No | Advanced | Mono | 28 | No | No | No | No | No |  |
| 2 | 59 | F | Ischemic stroke | No | Advanced | Mono | 42 | No | Yes (targeted, RT) | No | Yes | Yes | No AF during event |
| 3 | 79 | M | PE | No | Advanced | Mono | 27 | No | No | No | Yes | Yes |  |
| 4 | 45 | M | PE + DVT | No | Advanced | Combi | 35 | No | No | No | Yes | Yes |  |
| 5 | 74 | M | PE | Yes | Advanced | Mono | 36 | Yes (stroke) | Yes (targeted) | No | Yes | Yes |  |
| 6 | 68 | F | Ischemic stroke | No | Advanced | Mono | 29 | No | No | No | Yes | Yes | No AF during event |
| 7 | 36 | M | PE | No | Advanced | Mono | 26 | No | Yes (targeted) | No | Yes | No |  |
| 8 | 87 | M | Ischemic stroke | No | Advanced | Mono | 30 | No | No | No | Yes | Yes | No AF during event |
| 9 | 52 | F | DVT | Yes | Advanced | Combi | 29 | No | No | No | Yes | Yes |  |
| 10 | 67 | M | MI | No | Advanced | Mono | 26 | No | Yes (RT) | No | Yes | Yes |  |
| 11 | 52 | F | PE | Yes | Advanced | Combi | 31 | No | Yes (targeted+ RT) | No | No | No |  |
| 12 | 76 | M | PE | No | Advanced | Mono | 30 | No | No | No | Yes | Yes | Hospital admission during TE occurrence due to myocarditis and myositis |
| 13 | 70 | M | PE + DVT | Yes | Advanced | Combi | 28 | Yes (DVT) | No | LMWH | Yes | Unknown |  |
| 14 | 70 | M | Coronary artery disease requiring revascularization | Yes | Advanced | Mono | 31 | No | No | DOAC | Yes | No |  |
| 15 | 58 | F | Ischemic stroke | Yes | Advanced | Combi | 28 | No | Yes (targeted+ RT) | No | Yes |  | Hospital admission during TE occurence due to colitis grade 3.  No AF, but floating thrombus in aorta during event |
| 16 | 49 | M | PE | No | Advanced | Combi | 34 | No | No | No | Yes | Yes |  |
| 17 | 51 | F | Cerebral venous sinus thrombosis | Yes | Advanced | Combi | 31 | No | Yes (targeted) | No | No | No | Usage of 80mg prednison at time of TE occurrence (reason: hepatitis grade 3). Cerebral metastases demonstrated progression at time of TE occurrence. |
| 18 | 66 | M | TIA | No | Advanced | Mono | 28 | Yes (TIA) | No | Antiplatelet | Yes | Yes | No AF during event |
| 19 | 49 | F | PE + DVT | No | Advanced | Combi | 31 | No | No | No | No | No |  |
| 20 | 67 | M | PE | No | Advanced | Mono | 27 | Yes (TIA) | Yes (RT) | No | No | Yes | Hospital admission at time of TE occurrence |
| 21 | 75 | F | PE | No | Advanced | Combi | 21 | Yes (TIA) | No | Antiplatelet agent | No | No |  |
| 22 | 55 | M | PE | Yes | Advanced | Combi | 30 | No | No | DOAC | Yes | Yes |  |
| 23 | 64 | F | PE | No | Advanced | Combi | 22 | No | No | No | No | No |  |
| 24 | 60 | F | DVT | No | Adjuvant | Mono | 25 | Yes (DVT) | No | No | Yes | No | Medical history of heterozygote Factor V Leiden. |
| 25 | 63 | M | PE | No | Adjuvant | Mono | 40 | No | No | Antiplatelet agent | Yes | Yes | Hospital admission at time of TE occurrence |
| 26 | 71 | M | MI | No | Adjuvant | Mono | 26 | Yes (MI) | No | Antiplatelet agent | Yes | Yes |  |
| 27 | 50 | F | Ischemic stroke | No | Adjuvant | Mono | 43 | No | No | No | Yes | Yes | No AF during event |
| 28 | 74 | M | Ischemic stroke | No | Adjuvant | Mono | 27 | No | No | No | Yes | No | AF during event |

Abbreviations: AF, atrial fibrillation; ASA, acetylsalicylic acid; BL, baseline; BMI, body mass index; combi, ICI combination therapy; DOAC, direct oral anticoagulant; DVT, deep venous thrombosis; F, female; ICI, immune checkpoint inhibitor; LMWH, low molecular weight heparin; M, Male; MI, myocardial infarction; mono, ICI monotherapy; PE, pulmonary embolism; RT, radiotherapy; TE, thromboembolism; TIA, transient ischemic attack.

**Supplemental Table 2. Competing-risk regression for the occurrence of combined TE in the advanced disease cohort**

|  | Univariable | | | | Multivariable | | |
| --- | --- | --- | --- | --- | --- | --- | --- |
|  | N (%) | sHR | 95% CI | *P*-value | sHR | 95% CI | *P*-value |
| Age (≥ 65 years) | 155 (49) | 0.88 | 0.39-2.01 | 0.763 |  |  |  |
| Sex (female) | 131 (42) | 1.13 | 0.50-2.58 | 0.769 |  |  |  |
| Smoking (ever vs. never) | 128 (41) | 0.74 | 0.32-1.71 | 0.481 |  |  |  |
| WHO (≥ 1) | 112 (36) | 0.92 | 0.39-2.18 | 0.843 |  |  |  |
| BMI | Continuous | 1.07 | 1.02-1.12 | **0.010*** | 1.07 | 1.02-1.12 | **0.007*** |
| LDH (> 1 ULN) | 125 (40) | 1.40 | 0.62-3.17 | 0.420 |  |  |  |
| Brain metastases | 66 (21) | 2.29 | 0.95-5.49 | **0.064*** |  |  |  |
| ICI combination therapy | 102 (32) | 2.38 | 1.04-5.41 | **0.040*** | 2.47 | 1.08-5.65 | **0.032*** |
| History of VTE | 27 (9) | 0.50 | 0.07-3.61 | 0.495 |  |  |  |
| Khorana risk score at treatment start (≥ 1) | 101 (32) | 0.72 | 0.28-1.83 | 0.486 |  |  |  |
| Recent surgery | 48 (15) | 0.60 | 0.14-2.52 | 0.485 |  |  |  |
| Recent hospitalization | 29 (9) | 0.98 | 0.22-4.33 | 0.982 |  |  |  |
| Anticoagulant therapy (any) | 67 (22) | 1.24 | 0.49-3.16 | 0.650 |  |  |  |

Abbreviations: BMI, body mass index; CI, confidence interval; ICI, immune checkpoint inhibitor; LDH, lactate dehydrogenase; sHR, subdistribution hazard ratio; TE, thromboembolism; VTE, venous thromboembolism; WHO, World Health Organization.

**Supplemental Table 3. Incidence of thromboembolic events and incidence rates in the advanced disease cohort**

|  | **Monotherapy**  **(n=213)** | **Combination therapy**  **(n=102)** |
| --- | --- | --- |
| **Number of events (%)** | | |
| ATE | 6 | 1 |
| VTE | 6 | 10 |
| Total TE | 12 | 11 |
| **Incidence rate (per 100 person years (95% CI))** | | |
| ATE | 2.3 (0.9-4.8) | 1.0 (0.05-4.9) |
| VTE | 2.3 (0.9-4.8) | 9.9 (5.0-17.7) |
| Total TE | 4.6 (2.5-7.8) | 10.9 (5.7-18.9) |

Abbreviations: ATE, arterial thromboembolism; CI, confidence interval; TE, thromboembolism, VTE; venous thromboembolism.
